# The association between celiac disease and eosinophilic esophagitis in children and adults

**DOI:** 10.1186/1471-230X-13-96

**Published:** 2013-05-30

**Authors:** Michael J Stewart, Eldon Shaffer, Stephan J Urbanski, Paul L Beck, Martin A Storr

**Affiliations:** 1 Division of Gastroenterology, University of Calgary, Calgary, Alberta, Canada; 2 Division of Gastroenterology, Dalhousie University, Halifax, Nova Scotia, Canada; 3 Department of Pathology, Faculty of Medicine, University of Calgary, Calgary, Alberta, Canada; 4 Division of Gastroenterology, University of Munich, Munich, Germany

**Keywords:** Celiac disease, Eosinophilic esophagitis, Endoscopy, Incidence, Epidemiology, Standardized incidence ratios

## Abstract

**Background:**

An association between eosinophilic esophagitis (EoE) and celiac disease (CD) has been suggested in the literature. Our aim was to confirm and quantify the association between these two diseases.

**Methods:**

All patients in a large Canadian city diagnosed with EoE or CD over a five-year period were identified. Standardized incidence ratios (SIRs) with 95% confidence intervals (CIs) were calculated.

**Results:**

Over the five-year study EoE was diagnosed in 421 patients and CD was diagnosed in 763 patients. The incidence of EoE ranged from 2.1 to 10.7 cases per 100,000 population. The incidence of CD ranged from 10.4 to 15.7 cases per 100,000 population. Among the EoE cohort, 83 (20%) cases of EoE and 245 (32%) cases of CD were diagnosed in pediatric patients. The incidence of EoE in the pediatric subpopulation ranged from 3.7 to 6.9 cases per 100,000 population. The incidence of CD in the pediatric subpopulation ranged from 9.5 to 22.7 cases per 100,000 population. The concomitant diagnosis of both EoE and CD was made in three patients, all of whom were pediatric males. The SIR for EoE in the CD cohort was 48.4 (95% CI = 9.73, 141.41) with a SIR for CD within the paediatric EoE cohort of 75.05 (95% CI = 15.08, 219.28).

**Conclusions:**

This study confirms the association between EoE and CD. However, this association may be limited to pediatrics where the risk of each condition is increased 50 to 75-fold in patients diagnosed with the alternative condition. The concomitant diagnosis of these conditions should be considered in pediatric patients with upper gastrointestinal symptoms.

## Introduction

Eosinophilic esophagitis (EoE) and celiac disease (CD) are distinct entities affecting the upper gastrointestinal tract, with different clinical and histopathological features. Although eosinophilic infiltration of the esophagus was first described in the late 1970s, it was not until 1993 that EoE was proposed as a distinct clinicopathologic syndrome [1]. Originally thought to be an uncommon condition, its incidence has dramatically risen over the past decade, recently estimated at 4.4 to 9.5 cases per 100,000 person-years [2,3]. When first described, EoE was felt to be a predominantly pediatric condition, however, it is now commonly diagnosed in adults as well as children [4]. There is a striking male predominance with 76% of adult and 66% of pediatric cases being diagnosed in males [5].

The pathogenesis of EoE is not completely understood but appears to involve an interplay between genetic factors and environmental exposures. An association between EoE and allergic disorders such as asthma, atopic dermatitis, and food and environmental allergies has been long established [6-10]. Genome wide association studies (GWAS) have identified a number of single- nucleotide polymorphisms (SNPs) that appear to confer susceptibility to EoE [11,12]. In one pivotal study, a SNP in the gene that codes eotaxin-3, a cytokine critical to eosinophil migration, was associated with susceptibility to EoE [11]. Another GWAS has implicated over-expression of the gene that codes for thymic stromal lymphopoietin (TSLP) as an important factor in disease development. Interestingly, TSLP is a cytokine that regulates inflammatory response and has been implicated in other inflammatory conditions including asthma, inflammatory arthritis, and atopic dermatitis [13-15].

Celiac disease is an autoimmune enteropathy that has been estimated to affect up to one percent of the general population, although many remain undiagnosed [16]. Recent incidence estimates range from 2 to 17.4 cases per 100,000 population [17-20]. The clinical features can be rather non-specific but malabsorptive features dominate in the form of diarrhea, iron-deficiency anemia and weight loss; in childhood there is failure to thrive and growth retardation [21,22]. While CD was also once considered a childhood disease, the majority of cases are now discovered in adulthood with an average age of diagnosis of 46 years [21].

The development of CD is triggered by enteric exposure to the protein composite gluten in genetically predisposed individuals. The implicated genes at the HLA-DQ2 and/or DQ8 gene loci are found in 95 percent of people with celiac disease [23-25]. Recent genome wide studies have identified genes that convey overlapping susceptibility for a number of immune mediated conditions, including CD, type 1 diabetes, and Crohn’s disease [26]. To date, there have been no SNPs identified that would suggest a common genetic basis for EoE and CD. Furthermore, the frequency of HLA alleles associated with CD are no more common in adults with EoE than the general population [27].

The apparent absence of a genetic connection between EoE and CD is especially interesting given a clinical association that has been suggested in recent years. The first such reports were case studies of pediatric patients concomitantly diagnosed with both EoE and CD [28-31]. Two retrospective, cohort studies from Australia subsequently found a higher than expected prevalence of EoE among pediatric patients with CD. Leslie et al. [32] reviewed the medical records of 250 children with histologically confirmed CD over a seven-year period; 10 had been concurrently diagnosed with EoE. In a similar study, Ooi et al. [33] found that 7 of 221 children with CD had also been diagnosed with EoE. The estimated prevalence of EoE in these two pediatric CD cohorts was 4.0% and 3.2%, respectively. This is markedly higher than the 0.009% prevalence rate (0.9 per 10,000) reported for EoE in the general pediatric population [34]. Thompson et al. [35] recently reported on a cohort of 1142 adult and 297 pediatric CD patients presenting to a specialized CD referral centre in New York City and found an overall Standardized Incidence Ratio (SIR) for EoE of 16.0 using EoE incidence data from an Olmsted County population.

The incidence of autoimmune diseases of the gastrointestinal tract is increasing and despite excellent ongoing research the reason(s) remains unclear. As this is especially true of EoE and CD, we felt that it was of particular importance to confirm the association between EoE and CD using population-based pathology data. Given that the putative association has been mainly described in pediatric populations, we also sought to determine if this association extends to adults.

## Methods

We undertook a population-based review of all patients diagnosed with CD and EoE within a large Canadian centre. The Calgary Health Region (CHR) is the sole provider of all medical and surgical services for 1.2 million people, 300,000 of whom are younger than 19 years [36]. A centralized laboratory and pathology service processes all adult and pediatric endoscopic biopsy specimens and generates histopathology reports that are stored in a searchable database. A keyword search was used to identify all duodenal and esophageal biopsies obtained within the CHR between January 1, 2004 and December 31, 2008. These pathology reports were then reviewed to determine if histological evidence of EoE or CD was present. For this review we defined the pediatric subpopulation as all persons younger than 19 years of age.

Eosinophilic esophagitis is a clinicopathological diagnosis based on the presence of typical symptoms and an esophageal biopsy demonstrating greater than 15 eosinophils per HPF [5]. Cases of EoE within our study population were identified by histopathology reports stating that the biopsy was consistent with or diagnostic of EoE.

The diagnosis of CD was based on duodenal biopsies demonstrating typical histopathological features and classified using the modified Marsh criteria [37]. In general, biopsies demonstrating Marsh stage 2, 3a-c, and 4 lesions are diagnostic of CD. Marsh stage 1 biopsies are less specific and only considered to represent CD in the setting of a positive serological marker. (e.g. elevated anti-tissue transglutaminase). At the time of this study there where no formal requirements for pathology reporting of duodenal specimens, therefore, pathology reports that did not specify a Marsh stage were considered positive for CD when the pathologist commented that the biopsies were consistent with, or diagnostic of CD. Serologic confirmation of CD was obtained if pathologist comments were indeterminate (e.g. Marsh stage I lesions) and determined to represent a positive case of CD is a serologic marker was consistent with the diagnosis of CD and negative if the serology was normal or unavailable.

### Statistical analysis

Crude incidence rates were calculated using CHR population data for the years 2004–2008 [36]. Crude incidence rates were age- and gender-adjusted using the age and gender distribution of the 2006 Canadian population [38] as the reference population. This adjustment was performed in order to mitigate the effect of any regional variability in age and gender distribution.

To quantify a relationship between EoE and CD, SIRs with 95% confidence intervals (CIs) were calculated [39]. A SIR is an estimate of the true occurrence of disease in a population relative to what might be expected based on incidence rates in an average (“general”) population. The ratio comes from dividing the observed number diagnosed (the number of cases of a specific disease) within a reference population by the expected number within the general population. A SIR greater than unity (1.0) would indicate a higher than expected number of cases within the reference population as compared to the “general” population.

This study was approved by the Conjoint Health Research Ethics Board at the University of Calgary and is in agreement with the Declaration of Helsinki.

## Results

Over the five-year study period 421 patients were diagnosed with EoE. The age- and gender-adjusted incidence of EoE ranged from 2.1 cases per 100,000 population in 2004 to 10.7 cases per 100,000 population in 2008 (Table 1). As previously reported, over the same time period 763 patients were diagnosed with CD [17]. The age- and gender-adjusted incidence of CD ranged from 10.4 per 100,000 population in 2004 to 15.7 per 100,000 population in 2007 (Table 1).

**Table 1:** Incidence of celiac disease and eosinophilic esophagitis, per 100,000 population, age- and gender- adjusted, by year

	**2004**	**2005**	**2006**	**2007**	**2008**
Celiac disease					
Total population	10.4	10.7	13.0	15.7	14.0
Pediatric subpopulation	9.5	18.4	16.3	22.7	16.0
Eosinophilic esophagitis					
Total population	2.1	5.0	7.2	9.4	10.7
Pediatric subpopulation	6.6	6.9	3.7	6.3	4.6

**Figure 1:**
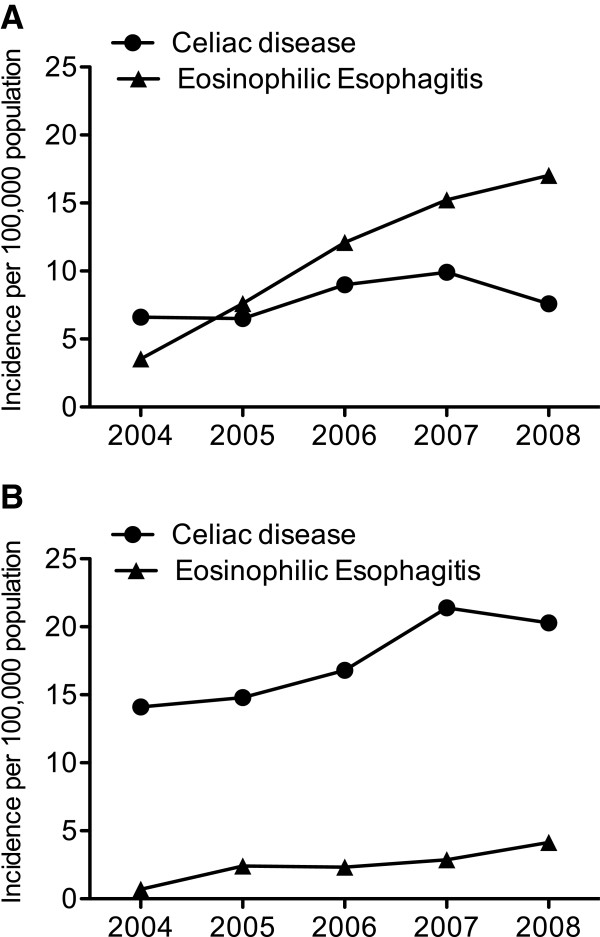
**The Incidence of Celiac disease and eosinophilic esophagitis.** Panel **A**) shows the incidence of both Celiac disease and eosinophilic esophagitis in males, per 100,000 population, age- and gender- adjusted, by year. Panel **B**) shows the incidence of Celiac disease and eosinophilic esophagitis in females, per 100,000 population, age- and gender- adjusted, by year.

When we looked at the gender distribution within the EoE and CD cohorts we found a significant male predominance for EoE with 82% (344) of patients being male. In contrast, within the CD cohort the opposite was true, only 32% (241) of patients were male (Figure 1).

Both conditions were commonly diagnosed in the pediatric age group. Among the EoE cohort, 83(20%) cases were diagnosed in patients younger than 19 years of age and within the CD cohort 245 (32%) cases were diagnosed in patients younger than 19 years of age. The age- and gender-adjusted incidence of EoE within the pediatric subpopulation ranged from 3.7 to 6.9 cases per 100,000 population. The age- and gender-adjusted incidence of CD within the pediatric subpopulation ranged from 9.5 to 22.7 cases per 100,000 (Table 1).

The diagnosis of both EoE and CD was made in three patients, all of whom were males in the pediatric age group. No adults were diagnosed with both conditions. The pertinent clinical, endoscopic and histological details are summarized in Table 2. Patient #1 was diagnosed with both conditions after presenting with a food bolus obstruction. At the time of EoE diagnosis the patient was on proton pump inhibitor (PPI) therapy and had a near-normal pH study. The diagnosis of CD was made by positive CD serology and Marsh III lesions on histology that normalized in response to a gluten-free diet. Patient #2 presented with abdominal pain and reflux and was diagnosed with EoE by the presence of the typical endoscopic and histologic findings. It is not clear if this patient was on a PPI or underwent a pH study. The diagnosis of CD was made by the presence of Marsh IIIb lesions and there was histologic normalization in response to gluten-free diet. Patient #3 presented with intermittent vomiting and at the time of EoE diagnosis he was on PPI therapy and had a near-normal pH study. The diagnosis of CD was made by positive serology and Marsh III lesions on histology. Repeat duodenal biopsies while on a gluten free diet showed mild villous blunting, however, CD serology normalized. None of the patients had evidence of gastric or duodenal eosinophilia on histology.

**Table 2:** Clinical, histological, and endoscopic findings in patients diagnosed with both eosinophilic esophagitis (EoE) and celiac disease (CD)

	**Patient # 1**	**Patient # 2**	**Patient # 3**
Symptoms	Food bolus obstruction and heartburn	Abdominal pain and reflux	Intermittent vomiting
Age at diagnosis	14 EoE / 14 CD	15 EoE / 15 CD	11 EoE / 11 CD
pH study	Normal	–	Normal
PPI	Yes	–	Yes
Other diagnoses	Asthma, allergies	–	Atopy, food allergies
Endoscopy findings			
Esophagus	Nodular mucosa and linear furrowing	Linear furrowing and adherent white plaques	Linear furrowing
Duodenum	“Scalloping” of the duodenum	Normal	Normal
Histology findings			
Esophagus	>15 EoE per HPF	>15 EoE per HPF	>15 EoE per HPF
Duodenum	Marsh III	Marsh IIIb	Marsh III
Gluten restriction	Normalized	Improved	Normalized

PPI, proton pump inhibitor.

The SIR for EoE within the pediatric CD cohort was 48.4 (95% CI = 9.73, 141.41). Conversely, the SIR for CD within the paediatric EoE cohort was 75.1 (95% CI = 15.08, 219.28). These SIRs imply that the concurrent diagnosis of EoE and CD in late childhood/adolescence occurred at a rate 48 to 75 times greater than predicted by incidence rates in the same population.

## Discussion

Celiac disease and EoE are two clinically, anatomically, and histologically distinct disorders of the upper gastrointestinal tract. An association between EoE and CD has been previously suggested. Contrary to what we expected to find in the current population-wide analysis that included a robust number of adults, this association may be solely a pediatric phenomenon. Although the overall number of patients with EoE and CD was low, no adults were diagnosed with both conditions, even though the majority of patients diagnosed with either condition were adults. Further, all three cases of EoE and CD were male. Although EoE is a predominantly male condition, CD is a predominantly female condition. In contrast to our findings, the Australian reports found that less than half of their patients with both diagnoses were males [32,33].

This study confirms the association between EoE and CD in pediatric patients. The prevalence of EoE in our five-year pediatric CD cohort was somewhat lower than reported in two previous studies despite our pediatric CD cohort being much larger than the previous studies (1.2% vs. 3.2% and 4.0%) [32,33]. It was, however, similar to the prevalence described in the New Your City CD cohort (0.97%) [35]. It is difficult to compare these results directly, or to other published prevalence rates, and it should be stressed that extracting prevalence estimates from a limited cohort identified by only five-years of incidence data undoubtedly underestimates the true prevalence of a disease within a population. Thus, we have presented SIRs to assess the association between EoE and CD. These SIRs provide a conservative estimate of the degree of association as they relay on a patient being diagnosed with both conditions within the five-year study period. Our somewhat lower rate of concomitant diagnosis does raise the possibility that coexistent EoE and CD might have been under-diagnosed in our study population, especially given the high incidence of autoimmune gastrointestinal illnesses in general within our population.

Importantly, the disease incidence rates we present in this paper compare favourably to those published from elsewhere in North America. In a recent study from Olmsted County, Ludvigsson and colleagues report the average CD incidence of 17.4 cases per100,000 person-years [20]. This is comparable to the CD incidence we present in this paper, which ranged from 10.4 to 15.7 cases per 100,000 population. A similar paper also from Olmsted County reported the incidence of EoE to be 9.45 cases per 100,000 person years [3]. This is equally comparable to the EoE incidence rates we report that demonstrate a steady increase from 2.1 to 10.7 cases per 100,000 population over the five-year study period.

A limitation in this study relates to the clinicopathologic diagnosis of EoE. The clinical information was not available and diagnosis was based solely on established histopathologic features of EoE. The presence of tissue eosinophilia can be seen in the setting of other gastrointestinal diseases, although usually eosinophil density is greater than 15 per HPF [40,41]. At the least, gastric eosinophilia was eliminated as all three patients diagnosed with EoE and CD had concurrent gastric biopsies that were normal. While we have demonstrated improvement or normalization of duodenal biopsies after initiating a gluten free diet, we cannot comment on the response of esophageal eosinophilia to gluten restriction.

The retrospective design of the current study did limit the availability of certain data which could have added to our analysis, including the total number and location of biopsies obtained from each patient, the symptoms that prompted the endoscopic investigation, and the response to therapy. It is conceivable that a patient undergoing an upper endoscopy for one condition may be more likely to be indecently diagnosed with another condition. It is also possible that pediatric gastroenterologists perform more routine biopsies of normal appearing mucosa than their adult colleagues. Both of these issues could serve to generate detection bias when it comes to the diagnosis of both conditions and emphasizes the need of future prospective studies.

Another limitation of this study is that we only assessed data over a five-year period. We were unable to determine if any of the patients had a previous histological diagnosis of CD or EoE prior to 2004 or from another jurisdiction. This issue could potentially result in an overestimation of the true incidence, however, it would also serve to underestimate the degree of disease association.

## Conclusion

The incidence of autoimmune diseases of the gastrointestinal tract is increasing. Although no genetic link between CD and EoE has been identified, this population-based study confirms the association between EoE and CD. The estimated risk of each condition is increased 50 to 75-fold in those pediatric patients diagnosed with the alternative condition. Furthermore, our data suggest that this significant association may be limited to the pediatric age group; no adults diagnosed with both conditions were identified. This unexpected finding highlights the urgent need for prospective studies in both children and adults. The major implication of this study is that the concomitant diagnosis of these conditions should be considered in pediatric patients with upper gastrointestinal symptoms.

## Abbreviations

(EoE): Eosinophilic esophagitis; (CD): Celiac disease; (SIRs): Standardized incidence ratios; (GERD): Gastroesophageal reflux disease; (HPF): High power field; (GWAS): Genome wide association studies; (SNPs): Single- nucleotide polymorphisms; (CHR): Calgary Health Region; (PPI): Proton pump inhibitor.

## Competing interests

The authors declare that they have no competing interests.

## Authors’ contributions

MJS: Study design, data analysis and interpretation, drafting of the manuscript. Dr. MJS has approved the final draft manuscript submitted. ES: Study concept and design, critical revision of the manuscript. Dr. ES has approved the final draft manuscript submitted. SJU: Data acquisition. Dr. SJU has approved the final draft manuscript submitted. PLB: Study concept and design, critical revision of the manuscript. Dr. PLB has approved the final draft manuscript submitted. MAS: Study concept and design, drafting and critical revision of the manuscript. Dr. MAS has approved the final draft manuscript submitted. All authors read and approved the final manuscript.

## Pre-publication history

The pre-publication history for this paper can be accessed here:

http://www.biomedcentral.com/1471-230X/13/96/prepub
